# Distribution of lower limb muscles may be associated with the progression of knee osteoarthritis and sarcopenia: a cross-sectional study

**DOI:** 10.3389/fmed.2026.1757121

**Published:** 2026-04-29

**Authors:** Fangming Yao, Zijun Zeng, Xuhui Yang, Bangning Gu, Jiawei Wu, Xiaoming He, Wei He, Qiushi Wei, Hongxing Huang, Mincong He

**Affiliations:** 1 The Third Clinical College of Guangzhou University of Chinese Medicine, Guangzhou, China; 2 The Third Affiliated Hospital of Guangzhou University of Chinese Medicine, Guangzhou, China; 3 Guangdong Research Institute for Orthopedics and Traumatology of Chinese Medicine, Guangzhou, China

**Keywords:** distribution of lower limb muscles, osteoarthritis, knee, radiography, ROC curve, sarcopenia

## Abstract

**Objective:**

This study aimed to delineate and segment the distribution of lower limb muscles based on full-length anteroposterior digital radiography (DR) of patients with varus knee osteoarthritis (vKOA), to quantitatively assess regional muscle abundance, and to explore its associations with vKOA progression and the risk of sarcopenia (SP).

**Methods:**

A total of 79 vKOA patients (124 lower limbs) admitted to the Joint Center of the Third Affiliated Hospital of Guangzhou University of Chinese Medicine between January 2023 and January 2024 were enrolled according to predefined inclusion and exclusion criteria. Inclusion criteria were: knee pain within the past month, age ≥ 50 years, morning stiffness < 30 min, and radiographic findings of joint space narrowing, subchondral sclerosis and/or cystic changes, and osteophyte formation. Exclusion criteria were: valgus KOA or other forms of arthritis, concomitant diseases affecting lower limb function, lower limb deformities of other etiologies, and inability to comply with data collection. Demographic and clinical information, including sex, age, height, weight, body mass index (BMI), diabetes, and hypertension, were collected. From DR images, muscle area indices were extracted from five anatomical sites: femoral lateral muscle, femoral medial muscle, tibial medial muscle, crural interosseous muscle, and lateral fibular muscle. Varus KOA related parameters, including hip–knee–ankle angle (HKA), joint line convergence angle (JLCA), medial joint space width (MJSW), and lateral joint space width (LJSW), were recorded. Appendicular skeletal muscle mass index (ASMI) was obtained using dual-energy X-ray absorptiometry (DXA). Patients were categorized into SP with vKOA (SP-vKOA) and vKOA groups according to the Asian Working Group for Sarcopenia criteria, and into mild vKOA (Kellgren–Lawrence grade 1–2) and severe vKOA (grade 3–4) groups. Intergroup analyses were performed using the rank-sum test, independent-sample *t*-test, and chi-square test. Binary logistic regression was applied to assess associations between muscle area indices and vKOA severity or SP risk, while linear regression was used to evaluate correlations between muscle distribution ratios and varus deformity. Receiver operating characteristic (ROC) curves were constructed to evaluate diagnostic performance.

**Results:**

Binary logistic regression indicated that the tibial medial muscle area index (TMMAI) [odds ratio (*OR*) = 0.054, 95% confidence interval (*CI*): 0.010–0.302] and weighted average density value (WADV) (*OR* = 0.996, 95% *CI*: 0.993–0.998) were significantly associated with SP occurrence. The combined *ROC* curve yielded an area under the curve (*AUC*) of 0.852 (95% *CI*: 0.727–0.881, *P* < 0.001), with a maximum Youden index of 0.594, optimal cutoff of 0.411, sensitivity of 79.17%, and specificity of 80.26%. Age (*OR* = 1.081, 95% *CI*: 1.014–1.152, *P* < 0.05) and the femoral lateral muscle area index (FLMAI) (*OR* = 0.120, 95% *CI*: 0.021–0.697, *P* < 0.05) were significantly associated with vKOA progression. The combined *ROC* curve showed an *AUC* of 0.789 (95% *CI*: 0.709–0.869, *P* < 0.001), maximum Youden index of 0.479, optimal cutoff of 0.533, sensitivity of 76.47%, and specificity of 71.43%. The ratio of femoral medial muscle area index to femoral lateral muscle area index (FMMAI/FLMAI) correlated with varus deformity, being significantly negatively associated with HKA (*t* = –2.64, *P* < 0.05), significantly positively associated with JLCA (*t* = 4.477, *P* < 0.05), and significantly negatively associated with MJSW (*t* = –4.278, *P* < 0.05).

**Conclusion:**

In patients with vKOA, atrophy of the femoral lateral muscle and imbalance in femoral medial-to-lateral muscle distribution may contribute to vKOA progression, while atrophy of the tibial medial muscle may be linked to the development of SP. Targeted rehabilitation strategies focusing on specific muscle groups in vKOA patients may help alleviate disease progression and reduce the incidence of SP.

## 1 Introduction

Osteoarthritis (OA) is a chronic degenerative joint disorder and a leading contributor to disability worldwide, with an estimated 300–500 million people affected (1, 2). Among its subtypes, knee osteoarthritis (KOA) is the most prevalent and poses a major challenge to older adults by causing persistent pain, restricted mobility, and reduced quality of life (3). Pathologically, KOA is characterized not only by cartilage degradation, subchondral bone remodeling, and osteophyte formation, but also by alterations in periarticular muscles and nerves that may exacerbate weakness and pain. Sarcopenia (SP), first defined by Rosenberg in 1989, is recognized as a geriatric syndrome marked by age-related decline in skeletal muscle mass, together with reduced muscle strength and/or impaired physical function (4). Its prevalence is estimated at 10–16% globally among older adults (5), making it a rapidly emerging public health concern. Sarcopenia is associated with a range of adverse outcomes, including falls and fractures, loss of independence, frailty, and excess mortality (6).

A growing body of evidence suggests that bones, cartilage, and muscles are functionally interconnected, and that aging affects these tissues in parallel (7). Moreover, sarcopenia and KOA share multiple risk factors, including aging, obesity, and physical inactivity (8). Taken together, these findings indicate a potential but incompletely understood interplay between KOA and sarcopenia, underscoring the need for further investigation. KOA and SP share overlapping target populations, and the pathological progression of these two conditions may interact with one another (9).

Digital radiography (DR) is the most commonly employed imaging technique in routine clinical practice. Compared with computed tomography (CT) and magnetic resonance imaging (MRI), DR offers advantages in terms of speed, accessibility, and cost-effectiveness. Unlike CT or MRI, which typically focus on a single anatomical region, full-length anteroposterior (AP) DR images of the lower limbs enable comprehensive assessment of the hip, knee, and ankle joints on both sides. This approach provides critical information for evaluating lower limb alignment as well as the mechanical and biological function of the extremities. Additionally, muscle tissue exhibits clear radiographic visualization, with density values clearly distinguishable from those of bone and adipose tissue. Prior studies have demonstrated the feasibility of analyzing muscle contours using X-ray imaging (10, 11). Skeletal muscle may undergo atrophy due to aging or disuse, or be infiltrated by adipose tissue as a result of metabolic disturbances, leading to reductions in both muscle mass and radiographic density. Consequently, quantifying muscle projection area and skeletal muscle density on full-length DR images may serve as a reliable method for objectively assessing muscle quality. These previous studies provided the technical feasibility for the present work. Motivated by the need to preliminarily assess lower limb muscle mass and distribution in patients with varus knee osteoarthritis (vKOA) to evaluate their current muscle status and potential risk of disease progression, this study employed DR to depict the coronal-plane projected area of lower limb muscles as a means to achieve this goal.

Building on this rationale, the present study aimed to examine, using full-length AP digital radiographs of the lower limbs of patients with vKOA, the relationship between lower limb muscle distribution, the progression of vKOA, and the occurrence of SP. The results are expected to elucidate changes in lower limb muscle groups among vKOA patients and their association with sarcopenia, thereby providing evidence to guide the development of targeted rehabilitation programs that focus on specific muscle groups.

## 2 Materials and method

### 2.1 Study population and ethics

Between January 2023 and January 2024, patients diagnosed with vKOA and admitted to the joint center at the Third Affiliated Hospital of Guangzhou University of Chinese Medicine were sequentially enrolled. At admission, demographic and clinical data were obtained, including age, sex, height, weight, body mass index (BMI), and medical history. All subjects were evaluated for appendicular skeletal muscle mass index (ASMI) using dual-energy X-ray absorptiometry (DXA) (manufacturer: Hologic, United States; model: AXY-00409) and full-length AP digital radiographs of the lower limbs (manufacturer: Siemens, Germany, model: Multix Fusion Max). After following the predefined inclusion and exclusion criteria, 79 patients (124 lower limbs) were enrolled in the study. Forty five patients submitted bilateral lower limb data, which were evenly divided amongst the study groups. This study was approved by the Ethics Committee of the Third Affiliated Hospital of Guangzhou University of Chinese Medicine (approval no. PJ-KY20221227-018), and written informed consent was obtained from all participants. The inclusion criteria were (a) recurrent knee pain within the past month; (b) age ≥ 50 years; (c) morning stiffness lasting < 30 min; (d) radiographic evidence of joint space narrowing, subchondral sclerosis and/or cystic changes, and osteophyte formation, with normal lower limb alignment or varus deformity. The exclusion criteria were (a) valgus KOA; (b) diagnosis of rheumatoid arthritis, gouty arthritis, or other types of arthritis; (c) comorbidities affecting lower limb function, including history of ipsilateral lower limb fracture, ipsilateral hip disease, acute stroke, post-stroke hemiplegia, or sequelae of poliomyelitis; (d) history of tumors or other diseases resulting in ipsilateral lower limb deformity; and (e) hearing or speech impairments or inability/unwillingness to comply with clinical data collection.

### 2.2 Assessment of knee osteoarthritis

Patients were classified using both the Kellgren-Lawrence (K-L) grading system (12) and the diagnostic criteria for sarcopenia proposed by the Asian Working Group for Sarcopenia (AWGS, 2019) (13). The K-L system grades the radiographic severity of KOA on a five-point scale: grade 0, no radiographic features of KOA; grade 1, doubtful joint space narrowing with possible osteophyte formation; grade 2, definite osteophytes without joint space narrowing; grade 3, moderate joint space narrowing; and grade 4, severe joint space narrowing accompanied by subchondral sclerosis.

### 2.3 Assessment of sarcopenia

The diagnostic criteria for sarcopenia, as defined by the Asian Working Group for Sarcopenia (AWGS), include (a) handgrip strength < 28 kg for men and < 18 kg for women; (b) appendicular skeletal muscle mass index (ASMI) < 7.0 kg/m^2^ for men and < 5.4 kg/m^2^ for women, measured by dual-energy X-ray absorptiometry (DXA); and (c) gait speed < 1.0 m/s assessed over a 6-m walking test. For functional evaluation, participants completed the 6-m walk along a hospital corridor at their usual pace. The time taken was recorded with a stopwatch, and gait speed was subsequently calculated.

### 2.4 Patient stratification

In this study, two stratification approaches were employed: (a) patients were categorized into vKOA and SP-vKOA groups based on the presence or absence of SP in order to investigate the relationship between lower limb muscle distribution and SP occurrence among vKOA patients; and (b) participants were further classified into mild vKOA (K-L grade 1–2) and severe vKOA (K-L grade 3–4) groups to assess the association between muscle distribution and vKOA progression. Furthermore, correlations between muscle distribution ratios and varus deformity were evaluated across the entire cohort. Our study included both bilateral and unilateral lower-limb samples. To minimize potential bias arising from the inclusion of bilateral samples, we sought to ensure that patients providing bilateral limbs were evenly distributed across different groups. Among all participants, 45 patients contributed bilateral lower-limb samples, including 30 in the vKOA group and 15 in the SP-vKOA group. Chi-square analysis demonstrated no significant difference in the proportion of bilateral KOA between these groups (χ^2^ = 3.052, *P* = 0.081). Similarly, in the Mild vKOA group, 20 patients had bilateral involvement, compared with 25 in the Severe vKOA group. Chi-square testing again revealed no significant intergroup difference (χ^2^ = 0.235, *P* = 0.627). These findings indicate that bilateral KOA samples were evenly distributed across all groups, with no statistically significant differences between groups.

### 2.5 Mapping of lower limb muscle area

Lower limb muscle distribution was mapped on full-length AP digital radiographs of the lower limbs following the protocol described by Hwang et al. (14). Based on skeletal landmarks and anatomical delineation, muscles were divided into five compartments: femoral lateral muscle, femoral medial muscle, tibial medial muscle, crural interosseous muscle, and fibular lateral muscle. Femoral lateral muscle refers to the muscle compartment extending from the inferior margin of the greater trochanter to the lateral femoral epicondyle. Its lateral boundary is approximately delineated by the contour of the vastus lateralis muscle. Femoral medial muscle denotes the muscle region extending from the medial gluteal fold to the medial femoral epicondyle. The medial boundary is defined by the projected margins of the adductor muscle group, gracilis, sartorius, and vastus medialis at different anatomical levels. Tibial medial muscle is defined as the muscle compartment extending from below the medial tibial plateau to the distal tibia. Its medial boundary corresponds to the muscle belly projections of the gastrocnemius and soleus muscles. Crural interosseous muscle refers to the muscle mass located between the tibia and fibula, primarily comprising portions of the tibialis anterior, tibialis posterior, soleus, and gastrocnemius muscles that overlap on the coronal plane. Fibular lateral muscle describes the muscle compartment located lateral to the fibula, extending from the fibular head to the distal fibula. This region mainly includes the peroneus longus muscle and partial muscle bellies of the gastrocnemius. All images were standardized with a window width of 4,095 and a window level of 2,048, which were the preset standards applied during the examinations. Regions of interest (ROIs) were manually delineated using the “Polygon selections” tool in ImageJ under consistent conditions.

**FIGURE 1:**
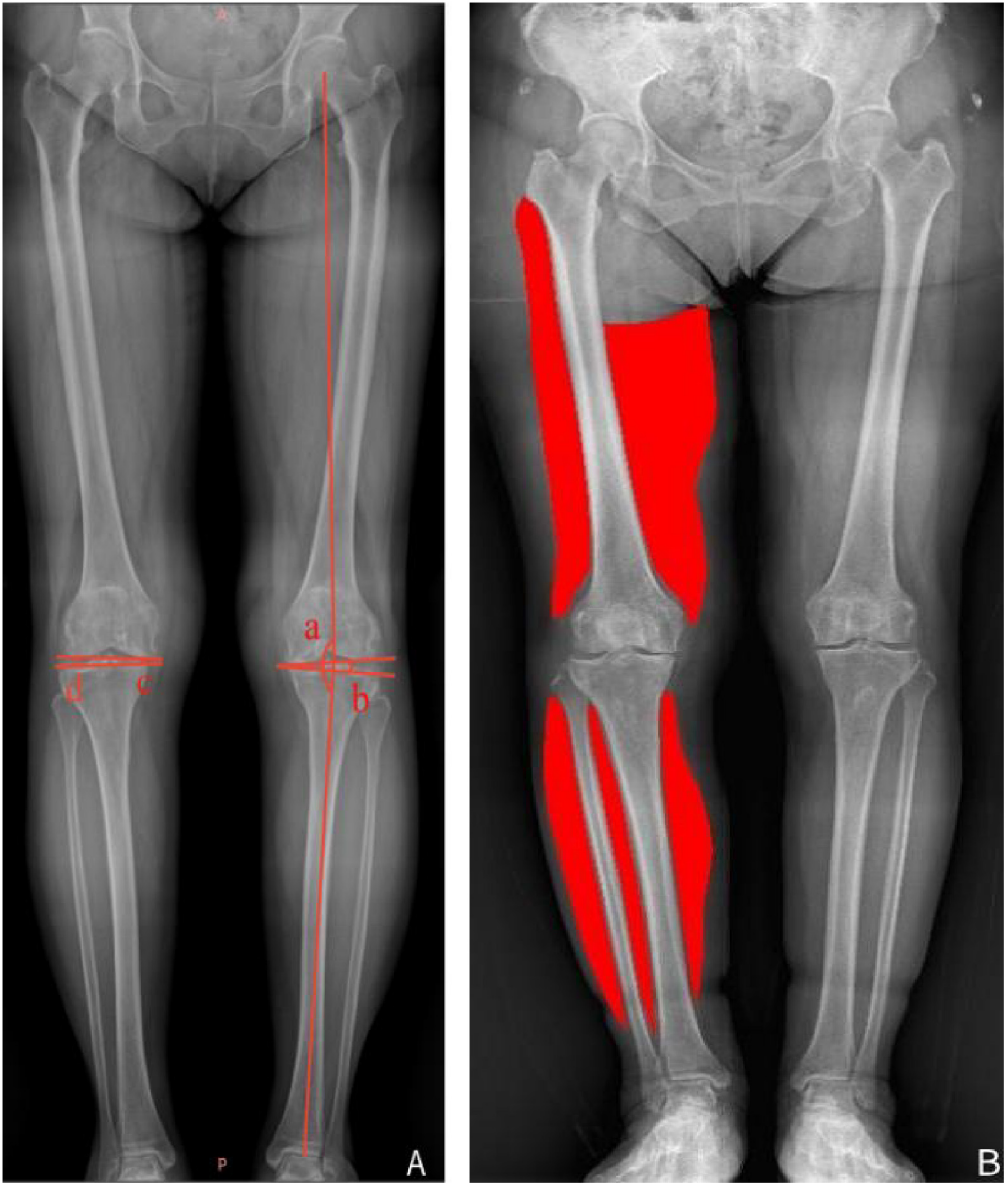
**(A)** Schematic representation of knee varus deformity: a, hip–knee–ankle angle (HKA); b, joint line convergence angle (JLCA); c, medial joint space width (MJSW); d, lateral joint space width (LJSW). **(B)** Mapping of lower limb muscle distribution was performed according to the method described by Hwang et al. (14).

For the femur, the superior boundary of the lateral compartment was defined by the inferior margin of the greater trochanter, and that of the medial compartment by the gluteal fold line. The inferior boundaries corresponded to the lateral and medial femoral epicondyles, respectively. Inner margins followed the femoral cortex, while outer margins were defined by the visible boundaries of longitudinal muscle fibers. For the lower leg, ROIs encompassed the visible portions of the soleus and gastrocnemius muscles, with inner margins along the tibial and fibular cortices and outer margins following the visible longitudinal muscle fiber boundaries (Figure 1).

The “Measure” function was used to determine the area and mean density of each ROI. A weighted average density value (WADV) was subsequently calculated across the five compartments. The WADV was calculated using the following formula:


$$W\,A\,D\,V=\frac{\sum_{i=1}^{5}A_{i}\times D_{i}}{\sum_{i=1}^{5}A_{i}}$$

where *A*_*i*_ denotes the muscle area of the *i*-th region, and *D*_*i*_ represents the corresponding muscle density value. To account for body size variability, muscle area indices (MAIs) rather than absolute muscle areas were computed according to the method of Gimigliano et al. (15). MAIs were defined as the ratio of each compartment’s muscle area to the patient’s body mass index (BMI). Accordingly, the femoral lateral MAI (FLMAI), femoral medial MAI (FMMAI), tibial medial MAI (TMMAI), crural interosseous MAI (CIMAI), fibular lateral MAI (FiLMAI), and total MAI (TMAI) were calculated. All measurements were performed independently by one experienced physician and subsequently reviewed by another physician to ensure accuracy. Intra- and inter-observer reliability for the measurement of lower limb muscle area and density were assessed by two dependent researchers, with all values indicating good to excellent reliability.

### 2.6 Assessment of knee varus alignment parameters

The hip–knee–ankle angle (HKA) was defined as the angle formed by the line connecting the centers of the hip, knee, and ankle joints. The joint line convergence angle (JLCA) was defined as the lateral angle between the tangents of the tibial plateau and the femoral condyles. Medial joint space width (MJSW) and lateral joint space width (LJSW) were defined as the shortest distances from the lowest points of the femoral medial and lateral condyles to the medial and lateral tibial plateaus, respectively (16).

### 2.7 Statistical analysis

All analyses were performed using SPSS version 26.0. Patients were stratified into SP-vKOA vs. vKOA groups and mild (K-L grades 1–2) vs. severe vKOA (K-L grades 3–4) groups. For normal distributed continuous variables, mean and standard deviation values are summarized, while, for non-normal variables, median and interquartile range (IQR) values are provided. Categorical variables were summarized using frequencies and percentages. Binary logistic regression was used to assess associations between regional muscle area indices and both vKOA severity and SP risk. Linear regression was applied to evaluate correlations between muscle distribution ratios and the degree of knee varus deformity. Receiver operating characteristic (*ROC*) curves were generated in GraphPad Prism version 9.5 to assess diagnostic performance, and area under the curve (*AUC*), 95% confidence intervals (*CIs*), sensitivity, specificity, and accuracy were calculated. Statistical significance was set at a two-sided *P* < 0.05.

## 3 Results

### 3.1 Baseline characteristics of participants in the vKOA and SP-vKOA groups

A total of 79 participants were enrolled in this study. Table 1 presents the baseline characteristics across groups, including age, sex, height, weight, hypertension, diabetes, and muscle area indices at different anatomical sites. Of particular note, patients with vKOA complicated by SP had significantly lower WADV (1209.96), FMMAI (4.68), TMMAI (1.13), and TMAI (10.79) compared with the vKOA group (1485.61, 5.28, 1.45, and 12.37, respectively; *P* < 0.05) (Table 1).

**TABLE 1:** Baseline characteristics and lower limb muscle distribution of vKOA group and SP-vKOA group.

Characteristics	Total (*n* = 124)	vKOA (*n* = 76)	SP-vKOA (*n* = 48)	*P* value
Height, Mean (SD)	157.16 (7.96)	156.95 (8.48)	157.48 (7.14)	0.723
Weight, Mean (SD)	55.76 (9.36)	56.05 (10.19)	55.30 (7.96)	0.666
CIMAI, Mean (SD)	1.05 (0.38)	1.07 (0.42)	1.03 (0.30)	0.550
WADV (IQR)	1377.29 (1194.58–1592.57)	1485.61 (1352.02–1709.80)	1209.96 (1078.71–1356.29)	< 0.001**
Age (IQR)	72.00 (66.00–77.00)	72.00 (67.00–77.00)	73.00 (64.00–76.00)	0.432
BMI (IQR)	22.86 (20.81–24.64)	23.14 (19.65–25.94)	21.95 (21.15–23.38)	0.181
FLMAI (IQR)	3.00 (2.45–3.52)	3.16 (2.50–3.62)	2.73 (2.39–3.28)	0.108
FMMAI (IQR)	4.96 (4.46–5.96)	5.28 (4.75–6.17)	4.68 (4.26–5.36)	0.005*
TMMAI (IQR)	1.34 (1.04–1.65)	1.45 (1.23–1.72)	1.13 (0.82–1.38)	< 0.001**
FiLMAI (IQR)	1.48 (1.37–1.74)	1.46 (1.35–1.73)	1.51 (1.40–1.74)	0.776
TMAI (IQR)	12.17 (10.41–13.45)	12.37 (11.25–13.55)	10.79 (10.08–13.15)	0.001*
Sex (male), n(%)				0.547
Yes	24 (19.35)	16 (21.05)	8 (16.67)	
No	100 (80.65)	60 (78.95)	40 (83.33)	
Hypertension, n(%)				0.228
Yes	108 (87.10)	64 (84.21)	44 (91.67)	
No	16 (12.90)	12 (15.79)	4 (8.33)	
Diabetes, n(%)				0.807
Yes	110 (88.71)	67 (88.16)	43 (89.58)	
No	14 (11.29)	9 (11.84)	5 (10.42)	

SD, standard deviation; IQR, interquartile range; vKOA, varus knee osteoarthritis; SP, sarcopenia; BMI, body mass index; FLMAI, femoral lateral muscle area index; FMMAI, femoral medial muscle area index; TMMAI, tibial medial muscle area index; CIMAI, crural interosseous muscle area index; FiLMAI, fibular lateral muscle area index; TMAI, total muscle area index; WADV, weighted average density value.

*P < 0.05;

**P < 0.001.

### 3.2 Risk factors associated with SP in patients with vKOA

Binary logistic regression analysis was conducted with the presence of sarcopenia (SP) in patients with vKOA as the dependent variable (“Yes” = 1, “No” = 0). Variables that showed statistically significant differences in Table 1 (*P* < 0.05) were entered into the model. Multicollinearity among the independent variables was assessed and found to be negligible. The analysis revealed that the tibial medial muscle area index (TMMAI) [odds ratio (*OR*) = 0.054, 95% confidence interval (*CI)*: 0.010–0.302] was an independent protective factor against SP. In addition, the WADV [*OR* = 0.996, 95% *CI*: 0.993–0.998] was significantly and inversely associated with the risk of SP (*P* < 0.05) (Table 2).

**TABLE 2:** Logistic regression analysis on risk factors for SP disease in vKOA patients.

Factors	Assign	*B*	*SE*	*Wald χ ^2^*	*P*	*OR*(95%*CI*)	
FMMAI	Continuous variable	-0.249	0.388	0.411	0.521	0.780	(0.364, 1.668)
TMMAI	Continuous variable	-2.915	0.876	11.077	0.001*	0.054	(0.010, 0.302)
TMAI	Continuous variable	0.324	0.252	1.66	0.198	1.383	(0.845, 2.265)
WADV	Continuous variable	-0.004	0.001	16.101	< 0.001**	0.996	(0.993, 0.998)
Constant		6.81	1.742	15.285	< 0.001**	907.214	

*OR*, odds ratio; SE, standard error; CI, confidence interval; FMMAI, femoral medial muscle area index; TMMAI, tibial medial muscle area index; TMAI, total muscle area index; WADV, weighted average density value.

*P < 0.05;

**P < 0.001.

### 3.3 *ROC* curve based on the multivariable binary logistic regression model

**FIGURE 2:**
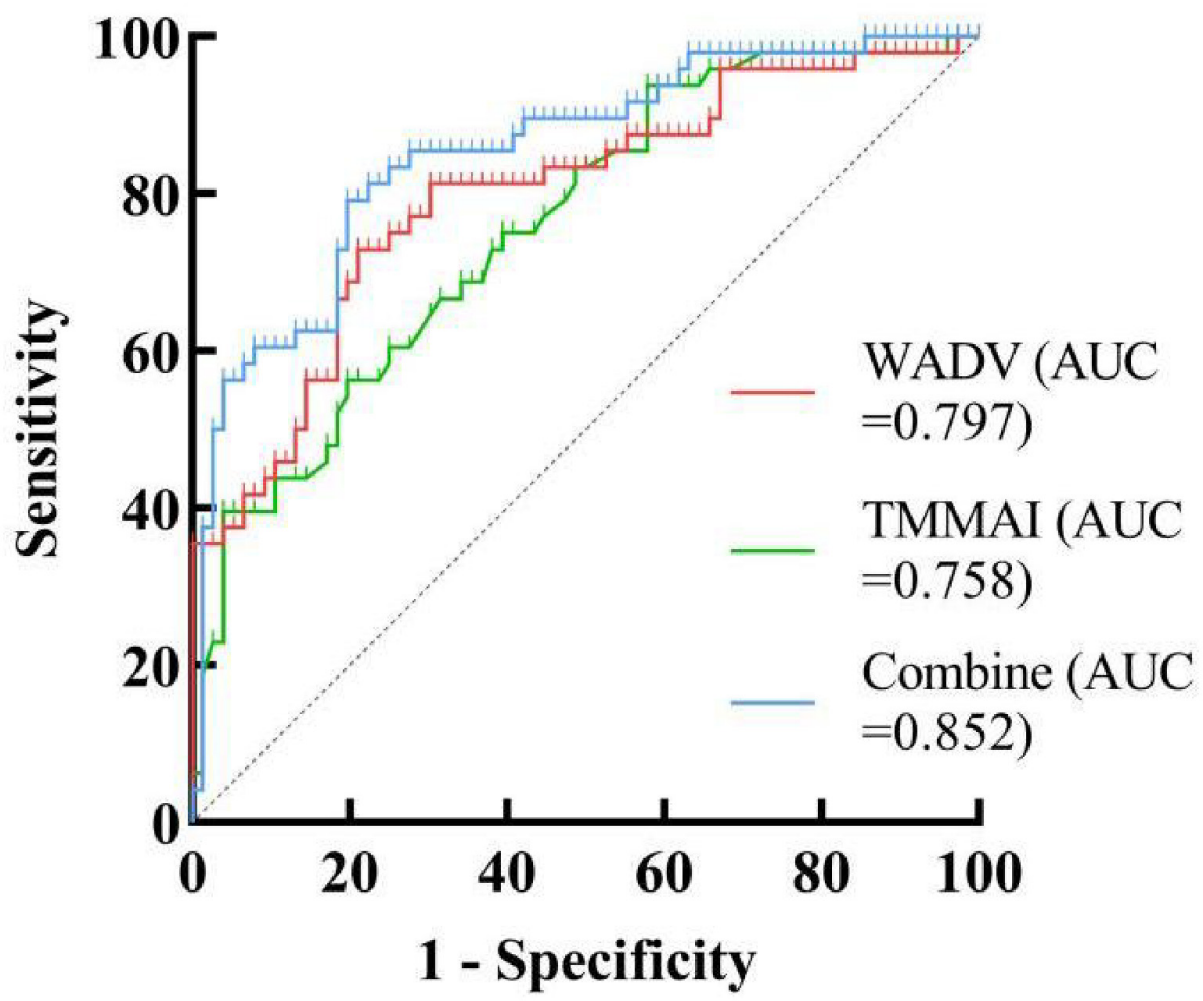
The receiver operating characteristic curve (*ROC*) for the diagnosis of SP-vKOA (Sarcopenia-Knee Osteoarthritis).

*ROC* curve analysis based on the multivariable binary logistic regression model indicated that TMMAI and WADV could be used for the diagnosis of sarcopenia in vKOA patients. The combined *AUC* was 0.852 (95% *CI*, 0.782–0.922; *P* < 0.001). The maximum Youden index was 0.594, with an optimal cutoff value of 0.411, corresponding to a sensitivity of 79.17% and a specificity of 80.26%, suggesting considerable diagnostic value (Figure 2).

### 3.4 Baseline information and clinical characteristics

A total of 56 knees were included in the mild vKOA group and 68 knees in the severe vKOA group. The severe vKOA group exhibited significantly shorter height compared with the mild vKOA group (*P* < 0.05). Conversely, the mild vKOA group had significantly lower age and BMI than the severe vKOA group (all *P* < 0.05). Regarding lower limb muscle distribution, the mild vKOA group showed higher values in CIMAI (1.13), FLMAI (3.35), FMMAI (5.48), FiLMAI (1.61), and TMAI (12.88) compared with the ever vKOA group (0.99, 2.59, 4.79, and 11.34, respectively; *P* < 0.05) (Table 3).

**TABLE 3:** Baseline data and lower limb muscle distribution of the mild vKOA group and the severe vKOA group.

Characteristics	Total (*n* = 124)	Mild vKOA (*n* = 56)	Severe vKOA (*n* = 68)	*P-*value
Age, Mean (SD)	71.65 (7.54)	69.34 (7.26)	73.54 (7.27)	0.002*
CIMAI, Mean (SD)	1.05 (0.38)	1.13 (0.36)	0.99 (0.38)	0.032*
WADV (IQR)	1377.29 (1194.58–1592.57)	1370.94 (1229.80–1515.68)	1427.68 (1156.08–1667.62)	0.735
Height (IQR)	157.00 (152.75–162.25)	158.00 (154.81–162.48)	155.50 (150.00–162.25)	0.107
Weight (IQR)	56.80 (50.00–61.12)	57.00 (50.00–60.44)	56.00 (50.00–64.00)	0.800
BMI (IQR)	22.86 (20.81–24.64)	21.95 (20.60–23.19)	24.03 (20.97–26.16)	0.008
FLMAI (IQR)	3.00 (2.45–3.52)	3.35 (2.95–3.98)	2.59 (2.30–3.20)	< 0.001**
FMMAI (IQR)	4.96 (4.46–5.96)	5.48 (4.58–6.59)	4.79 (4.38–5.33)	0.006*
TMMAI (IQR)	1.34 (1.04–1.65)	1.43 (1.07–1.68)	1.29 (1.03–1.50)	0.129
FiLMAI (IQR)	1.48 (1.37–1.74)	1.61 (1.42–1.76)	1.41 (1.31–1.65)	0.006*
TMAI (IQR)	12.17 (10.41–13.45)	12.88 (11.47–15.05)	11.34 (10.18–12.38)	< 0.001**
Sex (male), n(%)				0.941
Yes	24 (19.35)	11 (19.64)	13 (19.12)	
No	100 (80.65)	45 (80.36)	55 (80.88)	
Hypertension, n(%)				0.135
Yes	108 (87.10)	46 (82.14)	62 (91.18)	
No	16 (12.90)	10 (17.86)	6 (8.82)	
Diabetes, n(%)				0.339
Yes	110 (88.71)	48 (85.71)	62 (91.18)	
No	14 (11.29)	8 (14.29)	6 (8.82)	

vKOA, varus knee osteoarthritis; SP, sarcopenia; BMI, body mass index; FLMAI, femoral lateral muscle area index; FMMAI, femoral medial muscle area index; TMMAI, tibial medial muscle area index; CIMAI, crural interosseous muscle area index; FiLMAI, fibular lateral muscle area index; TMAI, total muscle area index; WADV, weighted average density value.

*P < 0.05;

**P < 0.001.

### 3.5 Binary logistic regression analysis of risk factors for vKOA progression

Binary logistic regression analysis was performed with the presence of severe vKOA as the dependent variable (“Yes” = 1, “No” = 0). Independent variables with statistically significant differences (*P* < 0.05) in Table 3 were included in the model. Multicollinearity among the independent variables was assessed and found to be negligible. The results indicated that age was a risk factor for vKOA progression (*OR* = 1.081, 95% *CI* 1.014–1.152, *P* < 0.05), whereas FLMAI served as a protective factor against vKOA worsening (*OR* = 0.120, 95% *CI* 0.021–0.697, *P* < 0.05) (Table 4).

**TABLE 4:** Logistic regression analysis on risk factors for disease progression in patients with vKOA.

Factors	Assign	*B*	*SE*	*Waldχ ^2^*	*P*	*OR*(*95%CI*)	
Age	Continuous variable	0.078	0.032	5.768	0.016*	1.081	(1.014, 1.152)
BMI	Continuous variable	0.086	0.071	1.46	0.227	1.090	(0.948, 1.254)
FLMAI	Continuous variable	–2.121	0.898	5.576	0.018*	0.120	(0.021, 0.697)
FMMAI	Continuous variable	–1.027	0.802	1.64	0.200	0.358	(0.074, 1.724)
CIMAI	Continuous variable	–1.182	0.801	2.174	0.140	0.307	(0.064, 1.476)
FiLMAI	Continuous variable	0.057	1.29	0.002	0.965	1.059	(0.084, 13.274)
TMAI	Continuous variable	0.714	0.679	1.107	0.293	2.042	(0.540, 7.722)
Constant		–5.144	6.979	0.543	0.461	0.006	

OR, odds ratio; SE, standard error; CI, confidence interval; BMI, body mass index; FMMAI, femoral medial muscle area index; FLMAI, femoral lateral muscle area index; FiLMAI, fibular lateral muscle area index; TMAI, total muscle area index.

*P < 0.05.

### 3.6 *ROC* curve analysis based on the multivariable binary logistic regression model

**FIGURE 3:**
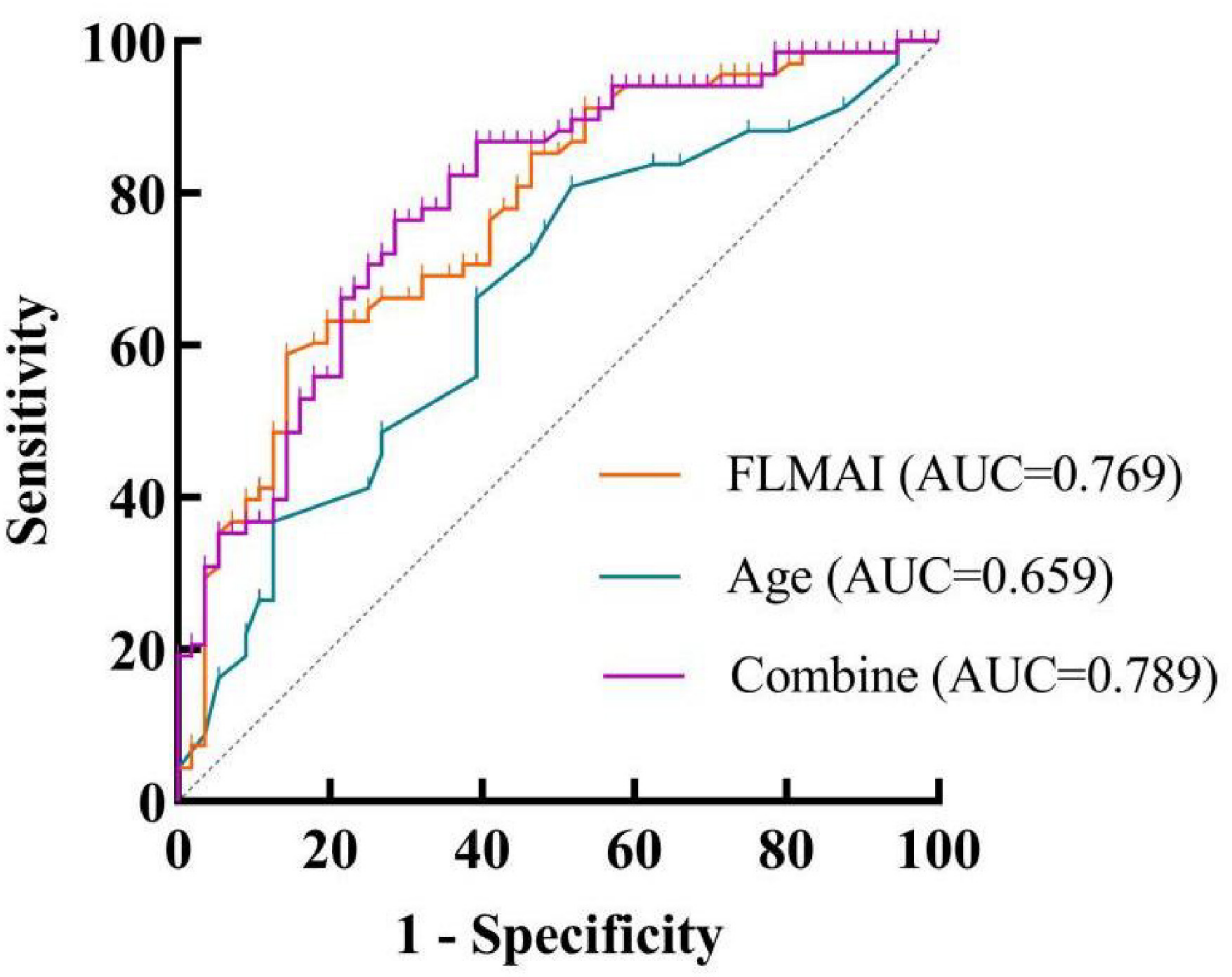
The receiver operating characteristic (*ROC*) curve for distinguishing between mild and severe cases of varus Knee Osteoarthritis (vKOA).

*ROC* curve analysis based on the multivariable binary logistic regression model demonstrated that age and FLMAI could be used to predict vKOA progression. The *AUC* was 0.789 (95% *CI* 0.709–0.869; *P* < 0.001). The maximum Youden index was 0.479, with an optimal cutoff value of 0.533, corresponding to a sensitivity of 76.47% and a specificity of 71.43%, indicating moderate diagnostic value (Figure 3).

### 3.7 Association of femoral medial-to-lateral and tibial medial-to-fibular lateral muscle area ratios with knee varus

In this study, the ratio of femoral medial-to-lateral muscle area (FMMAI/FLMAI) was employed to assess the balance between medial and lateral musculature of the femur and knee joint. Likewise, the ratio of tibial medial to fibular lateral muscle area (TMMAI/FiLMAI) was used to evaluate the balance of lower-leg muscle groups. To our knowledge, this study is the first to propose these ratios as novel indicators. Although no established numerical gold standard exists, intergroup comparisons provide a means to examine shifts in muscle distribution across different stages of vKOA progression.

**FIGURE 4:**
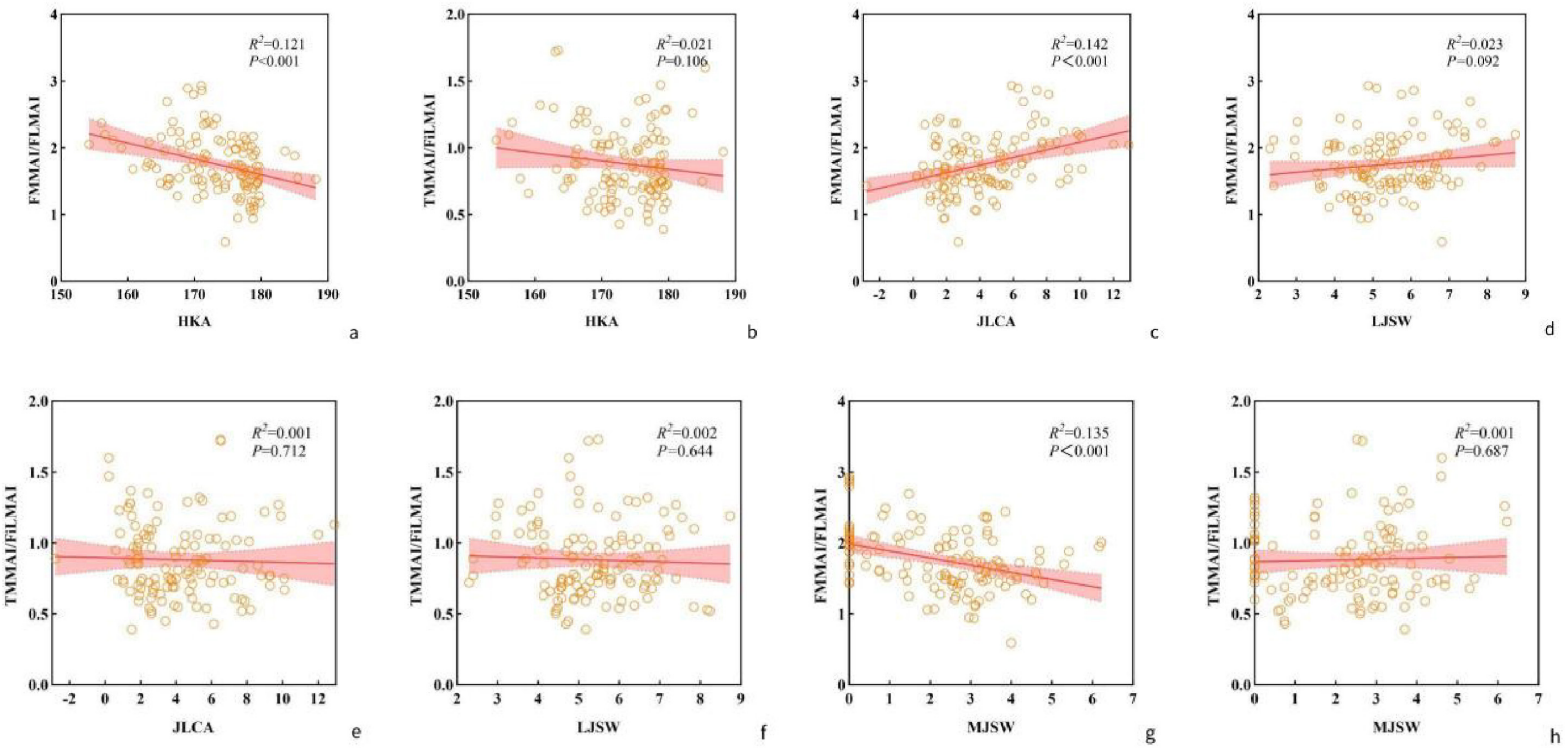
Correlation between the medial-to-lateral muscle area ratios of the lower limb and indicators of knee varus deformity.

HKA, JLCA, MJSW, and LJSW were used as observational parameters to reflect the degree of knee varus deformity. Linear correlation analyses were initially performed between these parameters and the FMMAI/FLMAI and TMMAI/FiLMAI. As shown in Figure 4, FMMAI/FLMAI was significantly associated with the severity of knee varus deformity (*P* < 0.05), whereas TMMAI/FiLMAI was significantly negatively correlated with MJSW (*P* < 0.05). No significant correlations were observed for the other parameters.

### 3.8 The linear regression analysis provided additional evidence supporting a significant association between the femoral medial-to-lateral muscle area ratio and knee varus severity

Building on the previous analyses, linear regression models incorporating potential confounders were performed to further assess these associations. HKA, JLCA, MJSW, and LJSW were specified as dependent variables. Confounders including age, height, and weight were entered into the models, while BMI was excluded due to multicollinearity. Variables reaching statistical significance in univariable analyses were subsequently included in multivariable linear regression. The multivariable results showed that, after adjustment for confounders, the femoral medial-to-lateral muscle area ratio was significantly negatively associated with HKA, positively associated with JLCA, and negatively associated with MJSW (all *P* < 0.05) (Table 5).

**TABLE 5:** Multivariate linear regression analysis of HKA, JLCA, MJSW, LJSW.

Dependent variables	Independent variables	*B*	*SE*	β	*t*	*P*
HKA	Constant	160.602	13.29		12.085	<0.001
	Age	–0.078	0.072	–0.094	–1.085	0.280
	Height	0.167	0.068	0.212	2.452	0.016*
	FMMAI/FLMAI	–4.489	1.233	–0.305	–3.642	< 0.001**
JLCA	Constant	–0.049	0.992		–0.049	0.961
	FMMAI/FLMAI	2.459	0.549	0.376	4.477	<0.001**
MJSW	Constant	4.634	0.559		8.284	<0.001
	FMMAI/FLMAI	–1.325	0.310	–0.361	–4.278	< 0.001**
LJSW	Constant	3.040	1.069		2.845	0.005
	Age	0.032	0.015	0.195	2.190	0.030*

HKA, hip-knee-ankle angle; JLCA, joint line convergence angle; MJSW, medial joint space width; *SE*, standard error.

*P < 0.05;

**P < 0.001.

## 4 Discussion

SP is a systemic skeletal muscle disorder characterized by a progressive loss of muscle mass and strength, with a high prevalence among older adults. Previous studies (5) have demonstrated that SP increases the risk of falls, cognitive decline, and mortality in this population. It substantially impairs quality of life in middle-aged and elderly individuals and frequently coexists with other age-related degenerative conditions, such as osteoporosis and KOA, thereby further elevating the risk of fractures and functional limitations (17–19). Based on a prior cross-sectional study conducted by our team, we hypothesized that the prevalence of SP is relatively high among KOA patients and may contribute to the progression of KOA pathology. In the present study, we examined differences in lower limb muscle distribution between vKOA and SP-vKOA patients, quantified the anteroposterior projection areas of lower limb muscles via imaging, and characterized the specific features of SP in the lower limbs of vKOA patients. These findings may provide valuable insights for the development of targeted exercise interventions aimed at improving functional outcomes and mitigating the progression of both vKOA and SP.

### 4.1 Atrophic features of SP maybe primarily reflected in the medial head of the gastrocnemius

Among vKOA patients, the most pronounced differences between those with and without sarcopenia (SP) are observed in the tibial medial muscle distribution. This region primarily comprises the well-developed medial head of the gastrocnemius, which originates from the medial femoral condyle and merges with the lateral head before inserting into the calcaneal tuberosity. The medial gastrocnemius contributes to knee flexion and plantarflexion and plays a critical role in maintaining posterior knee stability. Functioning as an antagonist to the anterior cruciate ligament (ACL), it also enhances sagittal-plane stability (20).

As the largest calf muscle, the gastrocnemius has been identified as an important target for predicting, diagnosing, and monitoring treatment outcomes of SP (21–23). Ultrasonographic assessments comparing elderly SP patients with age-matched controls have demonstrated significantly reduced gastrocnemius thickness in SP patients, with medial head thickness decline correlating with decreased muscle strength (24, 25). Cadaveric studies indicate that fat infiltration in the calf typically begins in the medial head of the gastrocnemius, progressing laterally toward the lateral head and the soleus (26). Atrophy of the gastrocnemius is also closely associated with flexion contractures in KOA, particularly in vKOA (27). Ultrasound-based measurements of the medial head of the gastrocnemius have been proposed for SP diagnosis (28). While DXA and bioelectrical impedance analysis (BIA) are commonly used for whole-body muscle assessments, local muscle evaluation is usually performed via ultrasonography to measure muscle thickness, cross-sectional area, and echogenicity (29). In contrast, the X-ray–based imaging approach used in this study provides a complementary perspective on overall muscle morphology and contour, which may enhance clinical diagnostic assessment.

### 4.2 Atrophy of the femoral lateral muscle is associated with vKOA progression

The femoral lateral muscle area index (FLMAI) was found to be significantly associated with vKOA progression. In our cohort, the femoral lateral region primarily encompassed the vastus lateralis, the most robust component of the quadriceps, which plays a key role in maintaining lateral knee stability. Previous studies have reported that the vastus lateralis in KOA patients exhibits a reduced fascicle angle compared with healthy controls, suggesting diminished patellar traction force (30). Levinger et al. (31) observed elevated levels of inflammatory kinases and cytokine proteins in the vastus lateralis of KOA patients, while muscle biopsies revealed marked reductions in quadriceps strength, type I fiber number, and satellite cell density, directly correlating with femoral lateral muscle atrophy and functional decline (32). Recent studies have also suggested that the phenotypic characteristics of satellite cells may determine the regenerative potential of skeletal muscle in patients with osteoarthritis (OA) (33). Beyond a reduction in satellite cell abundance, phenotypic alterations within satellite cell populations may represent an important mechanism underlying structural and functional muscle abnormalities in late-stage OA (34). Accordingly, therapeutic strategies targeting satellite cell abundance, phenotypic properties, and aberrant extracellular matrix deposition may serve as potential approaches to improve quadriceps strength and functional performance in this population. Additionally, prior research has documented significant reductions in vastus medialis muscle area in advanced versus early-stage KOA, consistent with our findings (35). Although all quadriceps components undergo some degree of atrophy during KOA progression, our results highlight the particularly pronounced changes in the vastus lateralis. Comparative assessments of individual quadriceps muscle strength (36) showed that, whereas the vastus medialis and rectus femoris largely retained strength comparable to controls, the vastus lateralis exhibited significant weakness. These observations indicate that the vastus lateralis is especially susceptible to atrophy and functional decline during KOA progression, emphasizing its relevance to disease severity, although the cross-sectional design precludes causal inference. From a biomechanical standpoint, the femoral lateral muscle group plays a pivotal role in maintaining frontal-plane stability of the knee joint. A reduction in lateral femoral muscle mass may compromise dynamic resistance to valgus collapse, thereby relatively increasing load transmission to the medial compartment and accelerating the development of varus malalignment. This redistribution of mechanical stress has been shown to aggravate cartilage degeneration and subchondral bone remodeling within the medial compartment, ultimately promoting the structural progression of knee osteoarthritis. This progression can be corrected by altering the lower limb mechanical axis through high tibial osteotomy (HTO) combined with computer-assisted navigation (37, 38). In this context, atrophy of the femoral lateral musculature may represent not only a consequence of pain-related disuse or reduced physical activity, but also a potential contributing factor to the progression of varus deformity.

### 4.3 Imbalance between medial and lateral muscles of femoral is associated with knee varus

The femoral medial-to-lateral muscle area ratio (FMMAI/FLMAI) was significantly linearly correlated with HKA, JLCA, and MJSW, indicating that the medial-lateral muscle balance is associated with the degree of knee varus in KOA patients. Atrophy of the vastus lateralis reduces lateral knee muscle strength, potentially causing relative laxity of the lateral collateral ligament and lateral supporting structures. This imbalance alters load distribution across the knee joint, resulting in an increased adduction moment from the medial femoral and medial gastrocnemius forces relative to abduction, placing greater stress on medial structures and exacerbating irreversible medial cartilage and subchondral bone damage. Studies have shown that in knees with varus deformity > 10°, medial supporting structures are shortened while lateral soft tissues are lax (39). Compared with healthy knees, OA knees exhibit increased vastus lateralis stiffness, correlating positively with the Western Ontario and McMaster Universities Osteoarthritis Index (WOMAC) scores (40). Spatial transcriptomic analyses indicate that KOA pathogenesis is linked to uneven load distribution across the knee. Quadriceps muscle imbalance may affect lower limb alignment and exacerbate unicompartmental OA, while targeted muscle strengthening and load redistribution can improve limb alignment and slow KOA progression (41, 42). Electromyography after high-tibial osteotomy (HTO) in varus KOA patients demonstrated reduced medial quadriceps and medial gastrocnemius adduction moments, improving joint alignment (43, 44).

Progressive knee varus is accompanied by internal rotation of the femur (45), which on anteroposterior projection may give the appearance of an increased femoral lateral muscle area and a reduced medial area. Despite this apparent enlargement, atrophy of the vastus lateralis was still evident. Femoral internal rotation also lengthens the patellar lever arm of the vastus lateralis, thereby reducing its contraction efficiency, altering patellar tracking, and increasing lateral patellofemoral joint loading—all of which may exacerbate the progression of varus KOA. Consistent with these biomechanical changes, previous studies have shown that varus KOA is frequently associated with lateral patellofemoral joint space narrowing. As varus deformity worsens, the tibial tubercle–trochlear groove (TT–TG) distance and patellar tilt both increase, reflecting medial displacement of patellar tracking and reduced mechanical advantage of the vastus lateralis in knee extension (46).

The discriminative ability of the models was evaluated using the *AUC* of the *ROC* curve, which represents the overall performance by integrating sensitivity and specificity across all possible thresholds. An *AUC* value of 0.5 indicates no discriminative ability, whereas an *AUC* value approaching 1.0 reflects excellent discrimination. *ROC* demonstrated that TMMAI and WADV exhibited good discriminative performance in predicting SP risk, with a combined model achieving an *AUC* of 0.852. This finding indicates that the integrated use of muscle-related indices provides high sensitivity and specificity in distinguishing individuals at high and low risk of SP, highlighting the potential clinical value of these parameters for early risk stratification and identification of SP. In addition, age and FLMAI showed moderate discriminative ability for predicting vKOA progression, with a combined *AUC* of 0.789. Although age alone demonstrated relatively limited diagnostic performance, its combination with FLMAI substantially improved the overall discriminative capacity. Notably, while the *AUC* of individual indicators were below 0.8, the combined models achieved clinically meaningful diagnostic performance, underscoring the advantage of multi-parameter integration in the risk assessment of complex musculoskeletal conditions, which represents an important conclusion of the present study.

This study has several limitations. Only patients with varus knee osteoarthritis (vKOA) were included, and the single-center design may introduce selection bias. Analysis was limited to anteroposterior digital radiographs without lateral views, which restricts assessment to a two-dimensional silhouette and does not fully capture three-dimensional muscle morphology. Although WADV was used as a surrogate measure of muscle quality, it does not directly reflect intrinsic tissue composition or functional capacity. The cross-sectional design also prevents determination of longitudinal relationships, and the observed associations between lower limb muscle characteristics and vKOA or sarcopenia progression cannot be interpreted as causal; they may represent either contributing factors or consequences of disease progression. More high-quality cohort or case-control studies incorporating lateral imaging and longitudinal follow-up are needed to better understand dynamic changes in lower limb muscle distribution and their functional implications.

## 5 Conclusion

Iin vKOA patients, vastus lateralis atrophy and imbalance of femoral medial-to-lateral muscles may be associated with vKOA progression, while medial tibial muscle atrophy may be related to SP development. These findings provide important guidance for targeted rehabilitation strategies to alleviate vKOA progression and SP onset. Clinical translation of these findings, including high-quality trials investigating targeted muscle rehabilitation interventions, represents a critical direction for future research.

## Data Availability

The raw data supporting the conclusions of this article will be made available by the authors, without undue reservation.
